# Role of *Anonychium africanum* (Plantae, Fabaceae) in Metal Oxido-Inflammatory Response: Protection Evidence in Gonad of Male Albino Rat

**DOI:** 10.3390/antiox13091028

**Published:** 2024-08-24

**Authors:** Harrison A. Ozoani, Orish Ebere Orisakwe, Costantino Parisi, Loredana Assisi, Anthonet N. Ezejiofor, Kenneth O. Okolo, Chinna N. Orish, Rubina Vangone, Emidio M. Sivieri, Giulia Guerriero

**Affiliations:** 1 Word Bank Africa Centre of Excellence in Oilfield Chemicals Research (ACE-CEFOR), University of Port Harcourt, PMB, Choba, Port Harcourt 5323, Nigeria; ozoani.harrison@esut.edu.ng (H.A.O.); orish.orisakwe@uniport.edu.ng (O.E.O.); 2 Advanced Research Centre, European University of Lefke, Northern Cypus, Lefke, TR-10, Mersin 99101, Turkey; 3 Comparative Endocrinology Laboratories (EClab), Department of Biology, University of Naples, 80126 Naples, Italy; cparisi@iimcb.gov.pl (C.P.); assisi@unina.it (L.A.); r.vangone@studenti.unina.it (R.V.); sivieri.eclab@gmail.com (E.M.S.); 4 African Centre of Excellence for Public Health and Toxicological Research (ACE-PUTOR), University of Port Harcourt, PMB, Choba, Port Harcourt 5323, Nigeria; anthoneth.ezejiofor@uniport.edu.ng (A.N.E.); kenneth.okolo@esut.edu.ng (K.O.O.); 5 Department of Anatomy, Faculty of Basic Medical Sciences, College of Health Sciences, University of Port Harcourt, PMB, Choba, Port Harcourt 5323, Nigeria; chinna.orish@uniport.edu.ng; 6 Interdepartmental Research Center for Environmental (IRCEnv, CIRAm), Via Tarsia 31, 80135 Napoli, Italy

**Keywords:** *Anonychium africanum* (*Prosopis africana*), antioxidants, apoptosis regulator, heavy metals, hormones, *Prosopis africana* gonado-protective effects, male reproductive toxicity

## Abstract

Male fertility is strongly affected by the overexpression of free radicals induced by heavy metals. The aim of this study was to evaluate the potential antioxidant, anti-inflammatory, and gonado-protective effects of natural compounds. Biochemical and morphological assays were performed on male albino rats divided into five groups: a control group (water only), a group orally exposed to a metal mixture of Pb-Cd-Hg-As alone and three groups co-administered the metal mixture and an aqueous extract of the Nigerian medicinal plant, *Anonychium africanum* (*Prosopis africana*, *PA*), at three different concentrations (500, 1000, and 1500 mg/kg) for 60 days. The metal mixture induced a significant rise in testicular weight, metal bioaccumulation, oxidative stress, and pro-inflammatory and apoptotic markers, while the semen analysis indicated a lower viability and a decrease in normal sperm count, and plasma reproductive hormones showed a significant variation. Parallel phytochemical investigations showed that *PA* has bioactive compounds like phlobatannins, flavonoids, polyphenols, tannins, saponins, steroids, and alkaloids, which are protective against oxidative injury in neural tissues. Indeed, the presence of *PA* co-administered with the metal mixture mitigated the toxic metals’ impact, which was determined by observing the oxido-inflammatory response via nuclear factor erythroid 2-related factor 2, thus boosting male reproductive health.

## 1. Introduction

The adverse impact of heavy metals on the physiological systems of animals has been broadly reported [1,2,3,4]. Research over the years has shown that these substances are recognized as highly hazardous elements, particularly for their detrimental effects on human and animal health [2,3,5]. They have negative effects on reproductive tissues [6], which may be linked to the increase in testicular disorders [7]. Further, they have been shown to be implicated in delayed development and reduced fertility [8,9], testosterone (T) production, and the inducement of testicular morphologic damage [10,11]. Additionally, they have been associated with reduced sperm counts, elevated numbers of abnormal spermatozoa, testicular degeneration, and impaired testicular growth [12]. These adverse effects in male animals contribute to a decrease in both sperm quality and quantity [9] and may result in damaging genetic and epigenetic consequences affecting their fitness [13]. 

The harmful effects of heavy metals have been mostly evaluated through in vitro as well as in vivo studies using several term exposures to either one metal or to a combinatory mix [3,5,14,15,16]. Toxic metals, including lead, cadmium, mercury, and arsenic, are commonly present in our surroundings, found in various sources such as food, water, soil, and air. Exposure to these metals can have toxic effects on the testis, resulting in alterations to seminiferous tubules, testicular stroma, and a decrease in spermatozoa count, motility, viability, as well as aberrant spermatozoa morphology [17,18]. During exposure to metals, protective enzymes are activated or induced under oxidative stress, allowing the cell to keep its homeostasis. Nuclear factor erythroid 2-related factor 2 (Nrf2) plays a major role in the transcriptional activation of antioxidant genes via an antioxidant response element (ARE). Prior to its nuclear translocation, Nrf2 moved from the cytoplasm to the plasma membrane, according to immunocytochemistry and subcellular fractionation studies [19]. Intracellular transcription factors play key roles in regulating genes associated with cellular defense mechanisms. Notably, Nrf2, activator protein 1, and nuclear factor kappa B (NF-κB) are recognized for their involvement in cytoprotection [20]. Among these, Nrf2 stands out as a crucial mediator in modulating cellular stress levels. In quiescent cells, Kelch-like ECH-associated protein 1 (Keap1) interacts with Nrf2 in the cytoplasm, controlling its activity. However, upon exposure to oxidative stress, Nrf2 dissociates from Keap1, translocates to the nucleus, and induces the expression of cytoprotective genes [21]. This cascade leads to the activation of downstream antioxidant enzymes such as catalase (CAT), glutathione reductase (GR), superoxide dismutase (SOD), and glutathione peroxidase (GPx) [22]. Similarly, NF-κB transcription factors regulate a spectrum of genes involved in inflammatory responses, cell proliferation, and neoplastic transformation. These genes encompass various chemokines, cytokines, apoptotic regulators, adhesion molecules, and oncogenes [20]. Although heavy metals are implicated in influencing NF-κB activity, the precise molecular mechanisms remain elusive.

*Anonychium africanum* (Hughes and Lewis, 2022), also known as *Prosopis africana* (*PA*) or “Okpeye”, is one of the plants utilized in traditional medicine in south-eastern Nigeria. It is characterized by its dark rough bark, pale drooping foliage with small, pointed leaflets, and sausage-shaped fruit. Rich in carbohydrates, fiber, protein [23], potassium, magnesium, and significant amounts of essential amino acids and phytocompounds with antioxidative, anti-inflammatory, and neuroprotective activity against metal mixture in neural tissues [23,24], it is highly valued for its nutritional content. Fermentation further enhances the nutritional value of *PA* and its antioxidant properties, a practice commonly employed in Nigeria [25]. Whereas the literature seems to be inundated with studies on individual metal testicular toxicity, information remains sparse on the toxicity of heavy metal mixture. The present study has therefore been undertaken to evaluate the potential protective effects of *PA* against heavy metal mixture exposure on the oxido-inflammatory response in rat testicular tissues.

## 2. Materials and Methods

### 2.1. Collection of Anonychium africanum (Prosopis africana, PA) and Preparation of Crude Extract

African mesquite AM pods were harvested from Nsukka, Enugu State, Nigeria (Latitude: 6.857816/N 60 51′ 28.138″ Longitude: 7.411943/E 70 24′ 42.996″) and identified by Mr. Ozioko, Department of Botany, University of Nigeria, Nsukka, and were washed, sun dried for three days, and blended to a powdery form. A total of 100 g of the powder was mixed with 1000 mL of deionized water and shaken for 48 h [26]. The slurry was sieved and filtered through a Whatman filter paper No. 1. The extract was then separated and stored at 4 °C. The crude extract was processed in a methanol extraction method as previously described by Hossain et al. [27]. The resulting methanol extract was concentrated using a rotary evaporator, and the dried residue was subjected to quantitative phytochemical screening. The methanol fraction was subjected to a gas chromatography–mass spectrometry analysis.

### 2.2. PA Preparation for Analysis by Gas Chromatography-Mass Spectrometry (GC-MS)

The GC-MS analysis of the methanol extract was performed using the Thermo/Finnigan Surveyor System. For this, an Ion Trap mass spectrometer was used, coupled with an electrospray ionization (ESI) source. Data acquisition was performed and mass spectrometric data were evaluated using data analysis software (Xcalibur Qual Browser 3.1; Thermo Electron, San Jose, CA, USA). Sample preparation and chromatographic separation was carried out following the method reported in Orisakwe et al. [24] and in Bagewadi et al. [28]. 

### 2.3. Acute Toxicity Testing (LD50)

Acute oral toxicity (LD50) was performed following Lorke’s median lethal dose method [29].

### 2.4. Animal Ethics and Maintenance

All animal maintenance and experiments were conducted in accordance with the guidelines specified in the protocol sanctioned by the UNIPORT Research Ethics Committee with approval reference number UPH/CEREMAD/REC/MM72/093.

Male albino rats (n = 56), 6 weeks old and weighing 80–100 g, were housed in 421 × 290 × 190 mm plastic polymer cages. Ambient temperature for the rats was maintained at 25 ± 2 °C, 50 ± 10% relative humidity, and a 12 h light–dark cycle. Ad libitum access to standardized feed pellets was provided (Hybrid Feeds Ltd. (Kaduna, Nigeria), km 8, MFD, 4 October 2020, with an expiration date of 6 January 2023, NAFDAC No A9-0232). The feed composition included crude protein (15.5%), fat (3.6%), crude fiber (4.6%), calcium (1.1%), available phosphorus (0.40%), methionine (0.37%), lysine (0.77%), and metabolized energy (2550 kcal/kg). Additionally, the rats had access to deionized water. They were acclimatized in the UNIPORT Pharmacology Animal House for a period of 14 days.

### 2.5. Experimental Design and Dose Administration

**Figure 1:**
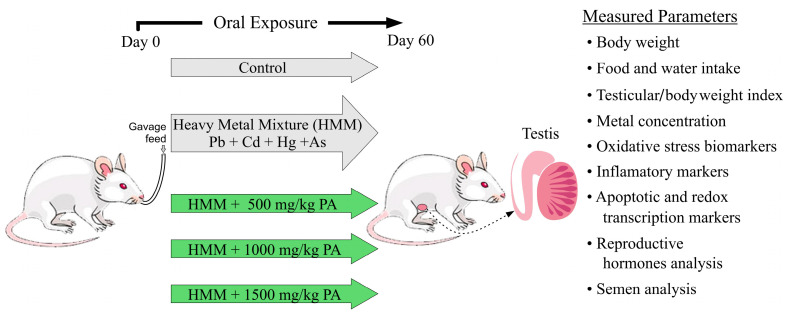
Experimental design: grouping, dose administration, and measured parameters.

Male albino rats were randomly divided into five groups, consisting of seven rats in each group. Both the untreated and treated rat groups received their respective, once daily, oral treatment doses by gavage for 60 days (Figure 1). The heavy metal mixture (HMM) used consisted of the following metals and dosages per kg of body weight: lead (II) chloride (20 mg/kg), mercury chloride (0.40 mg/kg), cadmium chloride (1.61 mg/kg), and sodium arsenite (10.0 mg/kg) [10,30,31].

Animal Experimental Groups:

- Group 1. Negative Control: This control group of rats was given deionized water orally once daily for 60 days.
- Group 2. Positive Control, HMM: This group received only the heavy metal mixture at the dose standards described above daily for 60 days.
- Group 3. HMM + *PA* (500 mg/kg): This groups received the same heavy metal mixture as the positive control but was treated with *Prosopis africana* aqueous extract at daily doses of 500 mg/kg body weight for 60 days.
- Group 4. HMM + *PA* (1000 mg/kg): This group received the same heavy metal mixture as the positive control but was treated with *Prosopis africana* aqueous extract at a daily dose of 1000 mg/kg body weight for 60 days.
- Group 5. HMM + *PA* (1500 mg/kg): This group received the same heavy metal mixture as the positive control but was treated with *Prosopis africana* aqueous extract at a daily dose of 1500 mg/kg body weight for 60 days.

### 2.6. Body Weight Measurement

Animals were reweighed using an Atom electronic balance at weekly intervals to monitor changes in body weight. Body weight changes at two-week intervals were used to recalculate the heavy metal mixture and *PA* doses to accommodate for changes in body weight. The percent body weight gain or loss was calculated as follows:

[Body weight on last day − body weight on day one]/body weight on day one × 100.

### 2.7. Measurement of Feed and Water Intake

A known weight (300 g) of feed and 200 mL of water were provided for each group of rats daily and the amounts consumed daily were recorded.

### 2.8. Necropsy, Tissues and Organ Collection and Processing

Animals in the five experimental groups were euthanized under mild pentobarbital anesthesia (50 mg/kg) at the end of 60 days of treatment. The epididymis of each rat was excised, and a semen analysis was performed. The testis were promptly excised from each male rat on a chilled dissection mat and washed in saline buffer (20 mM Tris–HCl, 0.14 M NaCl buffer, pH 7.4) once and then repeated. Organs were then weighed; one part of the testis was kept in Bouin’s solution for 24 h and then a histopathology analysis was performed. The testis (10% *w*/*v*) were homogenized in an ice-cold 50 mM Tris-HCl (pH 7.4) using a Potter-Elvehjem type glass-Teflon tissue homogenizer, sonicated (given 10 bursts, for 15 s each interval) using a PCI Analytics sonicator (model 500F, PCI Analytics, Thane, India) and then centrifuged at 3000× *g* at 4 °C for 15 min. Supernatants were then collected and stored at −20 °C for heavy metal mixture and biochemical assays, including tissue oxidative stress markers (CAT, SOD, GSH, GPX, MDA, and NO), ELISA assays for transcriptional factors (Nrf2 and NF-κB) and an apoptotic marker (caspase-3), and pro-inflammatory parameters (TNF-α and IL-6) [32].

### 2.9. Body Organ Index 

The relative organ weight was calculated as follows:

[specific organ weight/final rat body weight at last day] × 100

### 2.10. Metal Concentrations in Tissue Samples

The metal ion content was determined using one gram of each tissue sample as prepared according to the previously described procedure of Ozoani et al. [33]. 

### 2.11. Oxidative Stress Markers

Harvested rat testis were assayed for lipid peroxidation, which is marked by malondialdehyde (MDA). Adopting the protocol from Ohkawa et al. [34], tissue MDA levels were assayed spectrophotometrically. Nitric oxide (NO) was assayed using the Griess reaction [35]. Superoxide dismutase (SOD) activity was assayed by applying the previously described technique of Misra and Fridovich [36]. Reduced glutathione peroxidase (GPx) and glutathione (GSH) activity levels were assayed following the technique according to Guerriero et al. [37] and Rotruck et al. [38].

### 2.12. Measurement of Inflammatory Markers

The levels of nuclear factor kappa B (NF-κB; Cat no.: E-ELR0674, Elabscience Biotechnology Company, Beijing, China), interleukine-6 (Il-6; Cat. no.: E-EL-R0015, Elabscience Biotechnology Company, Beijing, China), and tumor necrosis factor alpha (TNF-α; Cat no.: RTA00-1, R&D Systems, Elabscience Biotechnology Company, Beijing, China) were detected in the testis of rats by enzyme-linked immunosorbent assay (ELISA) kits following the manufacturer’s instructions.

### 2.13. Measurement of Apoptotic and Redox Transcription Markers

The activity of caspase-3 (Cas-3) (Cat. no.: E-EL-R0160, Elabscience), and levels of nuclear factor erythriod 2-related factor 2 (Nrf2) (Cat. no.: E-EL-R1052, Elabscience) and Heme Oxygynase-1 (Hmox-1) (Cat. no.: E-EL-R0488, Elabscience) were assayed in the testis of rats from each of the control and treatment groups by enzyme-linked immunosorbent assay (ELISA) kits.

### 2.14. Reproductive Hormones Analysis

The reproductive hormones were analyzed in the plasma of male albino rats according to methods of Qiu et al. [39] for follicle-stimulating hormone (FSH); the methods of Frank and Rushlow [40] for luteinizing hormone (LH); the methods of Vanderpump et al. [41] for prolactin; and the methods of Guerriero et al. [42] for progesterone and testosterone. 

### 2.15. Semen Analysis

For the semen analysis, the epididymis was surgically removed, incised, and semen was aspirated into a dish with phosphate-buffered saline. After a 10 min incubation period, motility was assessed on a slide, categorizing sperm as active, sluggish, or immotile [43]. Viability was determined using an eosin–nigrosine stain, expressed in cell/mL [44]. The caudal epididymal sperm count was performed via hemocytometry [45], and morphology was examined after mixing with 2% eosin Y and incubation [46]. Morphological abnormalities were graded, and pH was measured using a pH meter, while viscosity was characterized as either highly viscous, semi- or slightly viscous, or non-viscous [47].

### 2.16. Statistical Analysis

Data were shown as the mean ± standard deviation. Statistical analyses were performed using SPSS (version 20 for Microsoft Windows, Albuquerque, NM, USA). The data were evaluated for normality and homogeneity by applying the Kolmogorov and Smirnoff test and the Levene test, respectively. Multiple variable comparisons were evaluated using a one-way analysis of variance using Microsoft Xlstat 2014. Tukey’s multiple range post hoc test was applied for comparing levels of significance between groups. Pandas was utilized in obtaining the descriptive statistical parameters for the rat testicular biomarkers. Correlation and regression analyses were performed to highlight the relationship between the protective action of *PA* and heavy metal-induced testicular oxidative complications and their pathophysiological changes as observed in the testis [48]. A multivariate analysis of variance consisting of principal component analysis and hierarchical cluster analysis (Euclidean distance measure) was applied to validate the curative action of *PA* on the oxidative damage to the testis [49]. Differences with a *p*-value of <0.05 were considered statistically significant.

## 3. Results

### 3.1. Phytoconstituents in Aqueous Extract of Anonychium africanum (Prosopis africana, PA)

Retention time (min), detected in the aqueous extract of *Prosopis africana* (*PA*) using gas chromatography–mass spectrometry (GC-MS), indicates compounds such as phlobatannins, flavonoids, polyphenols, tannins, saponins, steroids, and alkaloids, as shown by Orisakwe et al. [24]. 

### 3.2. Acute Toxicity Test of Prosopis africana Aqueous Extract 

The results of acute toxicity (LD50) after oral administration reveal that the *PA* aqueous pod formulation has an LD50 greater than 5000 mg/kg. Furthermore, no deaths were reported following the administration of the *PA* aqueous pod formulation at any of the doses administered. These findings suggest that this preparation possesses a wide therapeutic range and is relatively safe. 

### 3.3. Effect of Prosopis africana on the Body Weight and Absolute and Relative Weight of Testis of Male Albino Rats Exposed to HMM

The results in Table 1 reveal that rats treated with the HMM alone consumed less food and water when compared to the control group. However, when exposed to a combination of the HMM and the *PA* aqueous extract at the highest dose (*PA* 1500 mg/kg), rats exhibited values for feed intake and fluid intake similar to those of the control group. 

Furthermore, rats exposed to the HMM alone demonstrated a significant increase in the relative testicular weight when compared to the control group. In contrast, those exposed to a combination of the HMM and *PA* aqueous extracts at various concentrations showed a significantly lower relative testicular mass than the group exposed solely to the HMM, with values similar to those of the control group. 

Regarding body weight, rats in the control group gained more weight than rats in either of the treated groups, but the initial to final percent changes among the groups was not significant.

### 3.4. Prosopis africana Effect on Male Albino Rat Semen Exposed to Heavy Metal Mixture (HMM)

The sperm from rats exposed to the HMM exhibited lower viability, a decrease in normal sperm count, an increase in abnormal sperm count, and lower activity levels, with a high proportion of sluggish sperm and a significantly reduced overall sperm count compared to the control group (Table 2). In contrast, the sperm from rats exposed to a combination of the HMM and *PA* aqueous extracts at various concentrations demonstrated sperm indices similar to the control group, particularly with the highest concentration of the *PA* extract (1500 mg/kg). 

### 3.5. Prosopis africana Effect on Hormonal Profile of Male Albino Rats Exposed to HMM

**Figure 2:**
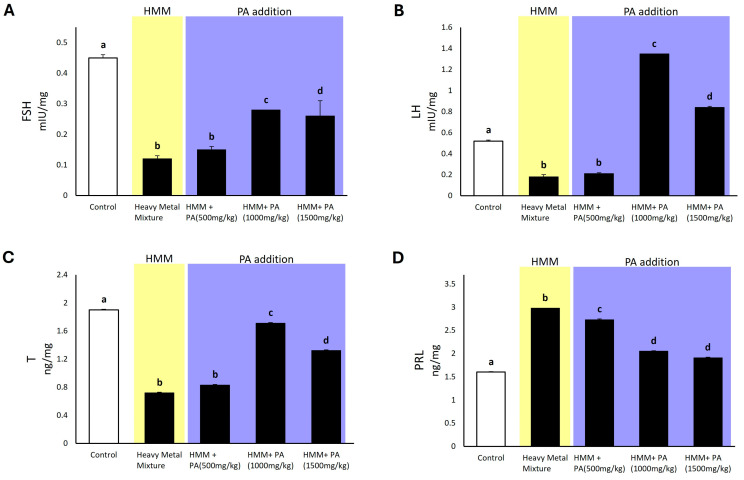
The impact of *Prosopis africana* (*PA*) on hormonal profile in plasma of male albino rats exposed to a heavy metal mixture (HMM) for 60 days. (**A**) the effect of *PA* on follicle-stimulating hormone (FSH). (**B**) The effect of *PA* on luteinizing hormone (LH). (**C**) The effect of *PA* on testosterone (T). (**D**) The effect of *PA* on prolactin (PRL). Values are mean ± SD, N = 7. Bars having the same letter notations (a, b, c, d) are not significantly different from each other (*p* ≥ 0.05).

Rats exposed to the HMM showed significantly decreased levels of follicle-stimulating hormone (FSH), luteinizing hormone (LH), testosterone (T), and prolactin (PRL) in the testicles when compared to the control group. Rats exposed to the HMM in combination with *PA* aqueous extracts exhibited significantly higher levels compared to those exposed to metals alone, showing a pronounced mitigation of the toxic metal effect. Regarding the FSH value, rats exposed to the HMM exhibited significantly lower values than the control. Notably, *PA* aqueous extracts at doses of 1000 mg/kg and 1500 mg/kg proved to be the most effective, as their presence in combination with the HMM led to a more substantial increase in FSH compared to exposure to metals alone (Figure 2A). Rats exposed to the HMM exhibited significantly lower LH levels (Figure 2B) than the control group, but in the presence of PA aqueous extracts at doses of 1000 mg/kg and 1500 mg/kg, rats exhibited a significant increase in LH levels compared to both the control group and the HMM only group. For testosterone, rats exposed to the HMM exhibited significantly lower values than the control. Remarkably, when exposed to the HMM together with *PA* aqueous extracts, the rats showed a significant increase in levels of testosterone compared to the rats exposed to metals alone (Figure 2C). Regarding prolactin, rats exposed to the HMM exhibited significantly higher values than the control. Interestingly, rats exposed to *PA* aqueous extracts at doses of 1000 mg/kg and 1500 mg/kg appeared to undergo a more pronounced effect in mitigating the toxic metal impact (Figure 2D).

### 3.6. Effect of Prosopis africana in Bioaccumulation of Heavy Metals in Rat Testis

**Figure 3:**
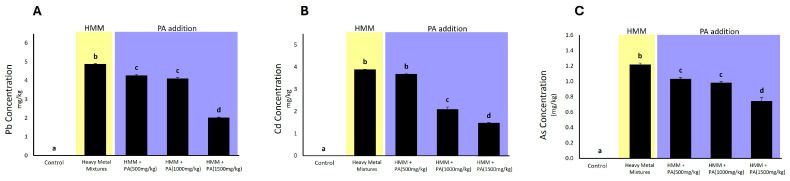
The concentration of heavy metal mixture (HMM) with and without *Prosopis africana* (*PA*) aqueous extracts on testicular tissue. (**A**) the concentration of lead (Pb) in testicular tissue exposed to HMM alone and the combination HMM and *PA*. (**B**) The concentration of cadmium (Cd) in testicular tissue exposed to HMM alone and the combination HMM and *PA*. (**C**) The concentration of arsenic (As) in testicular tissue exposed to HMM alone and the combination HMM and *PA*. Values are mean ± SD, N = 7. Bars having the same letter notations (a, b, c, d,) are not significantly different from each other (*p* ≥ 0.05).

Rats exposed to the HMM exhibited a significant accumulation of heavy metals in the testicular tissue compared to the control group. However, there was a significant reduction in heavy metal accumulation in the group exposed to HMM in combination with *PA* aqueous extracts (Figure 3) Moreover, as shown in Figure 3, there was a trend of decreasing bioaccumulation of lead, cadmium, and arsenic in a dose-dependent manner in rats exposed to the HMM in combination with *PA* aqueous extracts.

### 3.7. Effect of Prosopis africana on Oxidative Stress Markers of Male Albino Rat Testis Exposed to HMM

**Figure 4:**
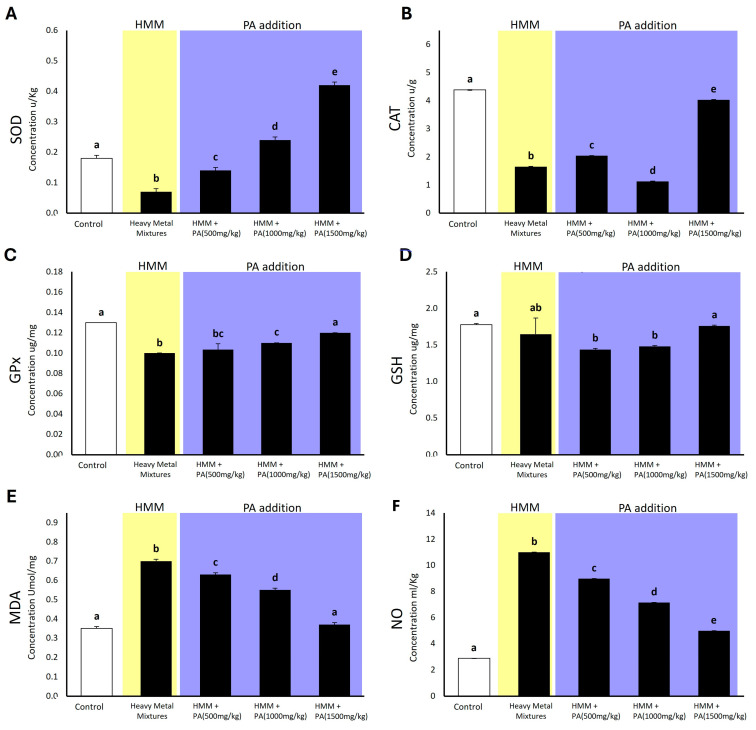
The impact of *Prosopis africana* (*PA*) on oxidative stress markers in male albino rats exposed for 60 days to a heavy metal mixture (HMM). (**A**) The effect of *PA* on SOD. (**B**) The effect of *PA* on CAT. (**C**) The effect of *PA* on GPx. (**D**) The effect of *PA* on GSH. (**E**) The effect of *PA* on MDA. (**F**) the effect of *PA* on NO. Values are mean ± SD, N = 7. Bars sharing the same letter notations (a, b, c, d, e) are not significantly different from each other (*p* ≥ 0.05).

The treatment of rats with the HMM significantly altered oxidative stress markers in the testis compared to the control group (Figure 4). Specifically, SOD and CAT levels were markedly reduced in the testis of rats exposed to the metal mixture. Simultaneous exposure to metals and *PA* aqueous extracts induced a dose-dependent increase in SOD levels, peaking at 1500 mg/kg of *PA*. Similarly, CAT levels reached their maximum at the highest concentration of 1500 mg/kg. GPx was significantly diminished in the testis of rats exposed to the HMM, but co-exposure to *PA* aqueous extracts resulted in an increase in GPx levels. Notably, the highest dose of 1500 mg/kg attained GPx levels similar to the control group. Contrarily, exposure to the metal mixture alone did not alter glutathione (GSH) levels in the rat testis. However, co-exposure with low (500 mg/kg) and medium (1000 mg/kg) concentrations of *PA* aqueous extracts caused a decrease in GSH levels. Intriguingly, the HMM with the highest *PA* dosage (1500 mg/kg) did not induce any changes in GSH levels. Levels of malondialdehyde (MDA) and nitric oxide (NO) were significantly elevated in the testicles of rats exposed to the heavy metal mixture. Conversely, the simultaneous exposure to the HMM and *PA* aqueous extracts resulted in a decrease in these levels, showing a dose-dependent trend and showing values similar to those of the control group.

### 3.8. Effect of Prosopis africana on Expression of Pro-Inflammatory Factors and Apoptotic and Transcriptional Factors in Male Albino Rat Testis Exposed to HMM

**Figure 5:**
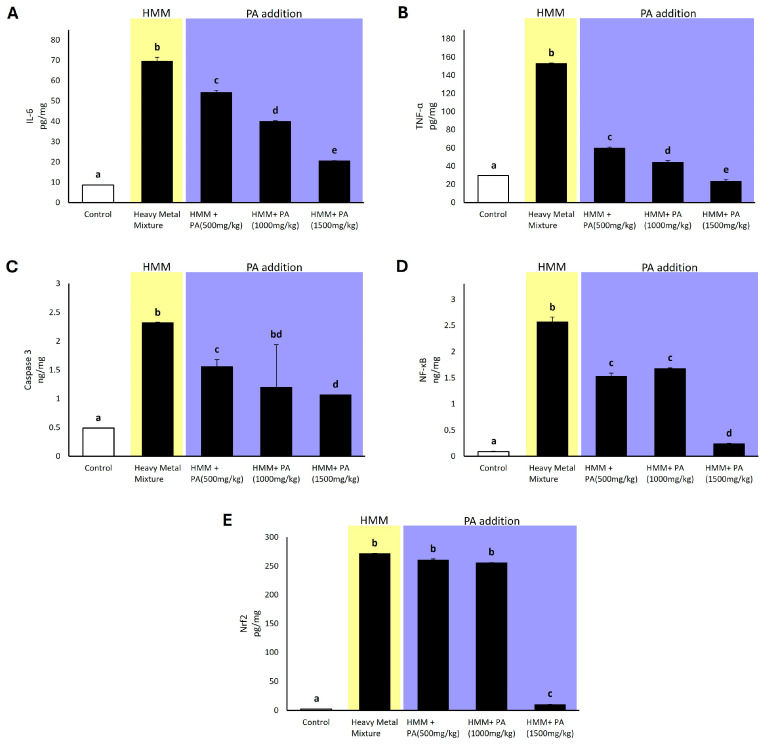
The impact of *Prosopis africana* (*PA*) on expression of pro-inflammatory factors and apoptotic and transcriptional factors in male albino rats exposed to a heavy metal mixture (HMM) for 60 days. (**A**) The effect of *PA* on interleukine-6 (IL-6). (**B**) The effect of *PA* on tumor necrotic factor alfa (TNF-α). (**C**) The effect of *PA* on caspase-3. (**D**) The effect of *PA* on nuclear factor kappa B (NF-κB). (**E**) The effect of *PA* on transcriptional factor Nrf2. Values are mean ± SD, N = 7. Bars sharing the same letter notations (a, b, c, d, e) are not significantly different from each other (*p* ≥ 0.05).

Rats exposed to the HMM showed significantly increased testicular levels of pro-inflammatory and apoptotic and transcriptional factors compared to the control group. Rats exposed to HMM in combination with *PA* aqueous extracts exhibited significantly lower levels compared to those exposed to metals alone, displaying a counteracting activity in reducing the effects of the toxic metals (Figure 5). Regarding the pro-inflammatory interleukine-6 (Il-6) and tumor necrotic factor-alfa (TNF-α), rats exposed to the HMM exhibited significantly higher values than the control. Specifically, the addition of the *PA* aqueous extract showed a dose-dependent trend in reducing their levels (Figure 5A,B). Regarding the apoptotic marker caspase-3, rats exposed to the HMM exhibited significantly higher values than the control. In the group with metal mixtures with *PA* aqueous extracts administered at doses of 1000 mg/kg and 1500 mg/kg, a significant decrease was induced compared to the HMM only group (Figure 5C). For the transcriptional factor NF-kappa B, rats exposed to the HMM exhibited significantly higher values than the control. Notably, when exposed to the HMM in conjunction with *PA* aqueous extracts, the rat testis showed a significant decrease in NF-kappa B levels compared to the group exposed to metals alone, with the lowest value observed at the dosage of 1500 mg/kg *PA* (Figure 5D). Regarding the transcriptional factor Nrf2, rats exposed to the HMM exhibited significantly higher values than the control. Interestingly, when rats were exposed to the HMM in combination with *PA* aqueous extracts, there was a significant decrease in Nrf2 levels compared to the group exposed to metals alone, indicating a suppressive activity, particularly at a *PA* dosage of 1500 mg/kg (Figure 5E).

## 4. Discussion

The purpose of this study was to assess the potential protective effect of *Anonychium africanum* (*Prosopis africana*, *PA*) against chronic testicular injury caused by intoxication from the heavy metal mixture of Pb-Cd-Hg-As. The co-treatment of testis with the extract of this plant appears to have significant effectiveness in mitigating the toxic effects of metals on testis, plasma, and semen exposed to a heavy metal mixture.

### 4.1. Chemical Characteristics and Relevant Activity of Prosopis africana

Firstly, the chemical profile of PA was evaluated using gas chromatography–mass spectrometry (GC-MS), revealing a concentration of phlobatannins, flavonoids, polyphenols, tannins, saponins, steroids, and alkaloids. Data from the experiments were already reported in our parallel study conducted on the neural system in the same rats [24]. Compounds such as flavonoids are organic chemicals present in a variety of plants, including PA [50,51,52,53,54,55], which have been shown to protect against oxidative injury [55,56]. Phenolic acids such as flavonoids possess antioxidant and anti-inflammatory activity [57,58,59,60,61]. Polyphenols [62,63], including resveratrol, catechin, epicatechin, naringin, and proanthocyanin, exhibit antioxidant and anti-inflammatory properties [64,65,66,67,68]. Alkaloids such as Sparteine and Ribalindine are known to be bivalent chelators and exhibit reactive oxygen species (ROS) scavenging, respectively, along with Ammodendrine and Aphyllidine [23,69,70,71,72,73,74]. *PA* is known to improve the expression of SOD, CAT, GPx, and NO by amino acids such as Citrulline [62,63]. These PA compounds appear to have played a decisive role in the biometric indices measuring the general health status of rats (see below) and the morphological data assessing reproductive health (Supplementary Figure S1). Our data, although innovative and encouraging, have shown limitations linked to the use of leaves, which have different phytochemical properties depending on their state of growth, and which may have had internal variations; a chemically tested product was not used, which, in the near future, will certainly allow for a more precise estimation.

### 4.2. Effect of Prosopis africana on the Body Weight and Absolute and Relative Weight of Testis of Male Albino Rats Exposed to Heavy Metal Mixture (HMM)

We observed that rats treated with a heavy metal mixture were characterized by weight loss, as reported by Cobbina et al. [31]. Several studies have linked exposure to chemicals to reductions in weight, water, and food intake [75], as well as the retardation of enzymatic activities, increased degradation of lipids and proteins, and degeneration of vital organs [76]. We also observed a significant increase in relative testicular mass compared to the control group, consistent with findings by Su et al. [77]. They reported that the coefficient of relative testicular weight in rats exposed to both individual metals and metal mixtures was higher than that of the control group, indicating a possible inflammatory response and edema induced in these organs. These observations were totally absent in the treatment involving the co-administration of the heavy metal mixture with *PA* aqueous extracts (Table 1) and with *PA* only. This last point is characterized by anti-inflammatory molecules such as humulone and resveratrol. Humulone has demonstrated significant anti-inflammatory activity by suppressing Cox-2 gene transcription in murine [57]. Resveratrol has been shown to modulate steroidogenic enzyme expression and the hypothalamus–pituitary–gonad axis, as well as alleviating oxidative stress in testicular tissues [78]. Both of these compounds could explain the counteracting effects of *PA* on the biometric and testicular indices due to metal exposure.

### 4.3. Effect of Prosopis africana in Bioaccumulation of HMM in Rat Testis

The administration of a quaternary metal mixture led to an increased accumulation of these metals in the testes of animals compared to the control group. Each of the metals used in our study mixture has been extensively evaluated for their detrimental effects on rat testis. In particular, it is recognized that Pb, Cd, Hg, and As can negatively impact sperm motility, while only Pb and Cd can negatively impact sperm viability and therefore total sperm count (see [6] for review). This accumulation, as suggested by previous studies [6,79,80], is assumed to originate from the intricate network of the heavy metal’s capacity to harm the blood–testis barrier via p38 mitogen-activated protein kinase signaling. This process involves the participation of heavy metal transporters and metallothioneins [81]. Our study shows that upon co-exposure with *PA*, there was a significant reduction in metal accumulation in the testis. This could be due to the chemical properties of *PA*, which could play a role in preventing or reducing heavy metal accumulation. Compounds such as chelators, antioxidants, enhancers of detoxification pathways, inducers of metallothioneins, and modulators of metal transporters protect or reduce metal bioaccumulation. Phytochelatins found in plants bind to the HMM, forming stable complexes that are less likely to be absorbed by animal tissues [82]. Additionally, their compounds exhibit antioxidant properties, scavenging free radicals to prevent cellular damage induced by the HMM [83]. Moreover, these kinds of compounds stimulate the expression of detoxification enzymes, facilitating the breakdown and elimination of the HMM from the body [84]. Lastly, the modulation of metal transporters regulates the uptake and distribution of the HMM in animal tissues, thereby decreasing their accumulation [85], as observed in our study.

### 4.4. Prosopis africana Affects Oxidative Stress Markers of Male Albino Rat Testis Exposed to HMM

The daily oral administration of *PA* to adult male rats effected a significant increase in the testicular expression of CAT, SOD, and GPx levels, along with a significant decrease in MDA and NO concentration compared to the metal mixture-treated rats. This improvement in the testicular antioxidative status of *PA*-treated rats may be the result of the high concentration of active antioxidants shown in Figure 4. The antioxidative effects of *PA* could be explained by the direct inhibition of lipid peroxidation and free-radical scavenging, or by the indirect increased activity of SOD and CAT, as observed with other natural compounds used to alleviate oxidative stress in male rats [86]. The present study shows that exposure to a quaternary metal mixture induced testicular oxidative stress, evidenced by reduced testicular CAT, SOD, and GPx levels, and elevated MDA and NO concentrations. These findings may be attributed to the generation of ROS, which deplete CAT, SOD, and GPx, ultimately leading to oxidative damage to the cell membrane, indicated by the increased MDA and NO concentrations [11,87,88]. Our results are supported by a previous study by Ozoani et al. [11], which showed that rats exposed to heavy metal compounds exhibit elevated testicular lipid peroxidation and a significant decrease in the levels of glutathione, CAT, SOD, and peroxidase. The testicular antioxidative status improvement by *PA* administration was evidenced by an increase in CAT, SOD, and GPx expression activity, along with the reduction in MDA and NO concentrations compared with the heavy metal mixture group. This finding may be attributed to the potent antioxidant components of *PA*, shown by Orisakwe et al. [24], that prevent cellular damage caused by oxidative stress in testis. Thus, the oral administration of *PA* protects against heavy metal toxicity via the mitigation of lipid peroxidation and decreased production of free-radical derivatives.

### 4.5. Prosopis africana Effect on Expression of Pro-Inflammatory Factors and Apoptotic and Transcriptional Factors in Male Albino Rat Testis Exposed to HMM

The consequences of induced oxidative stress through metal exposure are also observed in the adaptive response, involving both innate and acquired mechanisms. This leads to the activation of inflammatory and apoptotic pathways, along with damage to the antioxidant system. ROS and NO can both trigger the activation of TNFα, a pleiotropic cytokine capable of initiating various inflammatory and apoptotic pathways, such as NF-kappa B, IL-6, caspase-3, and caspase-9 [89]. Our findings revealed that metal exposure significantly increases the levels of TNFα, NF-kappa B, IL-6, caspase-3, and poly(ADP-ribose) polymerases, consistent with the findings of Kasperczyk et al. [90], Mognetti et al. [91], and Ozoani et al. [11].

As part of the adaptive cellular response, there appears to be an up-regulation of Nrf2, potentially aimed at safeguarding the testis from oxidative stress. Indeed, we noted an increase in Nrf2 expression following exposure to the HMM, a finding supported by similar studies [92]. However, these effects were absent upon co-exposure with *PA*. This finding can be attributed to the potent antioxidant properties of *PA*, which includes compounds such as resveratrol and catechin. The therapeutic effects of these components are associated with the modulation of the Nrf2 signaling pathway, known for its anti-inflammatory, antioxidant, hepatoprotective, neuroprotective, cardioprotective, renoprotective, anti-obesity, anti-diabetic, and anti-cancer properties [93]. Further studies will clarify, in detail, the mechanism of action of *Prosopis Africana* by Nrf2 which was particularly effective at a dose of 1500 mg/kg of PA.

### 4.6. Effect of Prosopis africana on Hormonal Profile of the Male Albino Rat Exposed to HMM 

The oral administration of a quaternary metal mixture to adult male rats caused a marked decrease in the expression of FSH, LH, and testosterone (T) levels, along with higher PRL levels compared to the control treatment. These results indicate that metals alter the function of the anterior pituitary, affecting LH and FSH production, as well as Leydig cells, which are involved in testosterone production. The reduced levels of LH and FSH may be attributed to disturbances in the negative feedback control of the hypothalamic–pituitary axis [86]. Furthermore, the impairment of pituitary function, such as LH secretion, may result from the impairment of cell membrane-mediated signaling pathways responsible for LH release [94]. The process of steroidogenesis in male rodents is induced by hypothalamic gonadotropin-releasing hormone (GnRH), which triggers the production and release of pituitary LH. LH then binds to the LH receptor (LHR) on the exterior of Leydig cells, stimulating testosterone synthesis. Consequently, the decline in testosterone concentrations is a rational outcome of the decrease in LH levels [95]. Thus, the reduction in circulating testosterone is hypothesized to stem from the direct toxic effect of the HMM on Leydig cells [96]. Similarly, Ozoani and colleagues [11] found that the quaternary metal treatment of adults significantly decreased FSH, LH, and testosterone levels compared with the control treatment [11]. Previous studies involving the simultaneous administration of sexual hormone effectors and natural compounds to adult males have shown improved pituitary and Leydig cell function and sex steroid receptor binding [86,97,98,99]. This improvement was reflected by increases in FSH, LH, and testosterone levels compared to the administration of sexual hormone effectors alone [86]. These effects were explained by the presence of many endogenous antioxidants in the natural compound, which reduce oxidative stress and ameliorate pathological changes in the testis [100]. Additionally, Farag and colleagues [101] reported that *Spirulina* administration to cadmium-intoxicated rats significantly increased testosterone levels compared with a cadmium treatment alone, highlighting the pivotal role of antioxidant molecules [101]. Thus, similarly, a protective effect of plasmatic sex hormones due to the co-administration of metals with *PA* aqueous extracts can be attributed to the antioxidant properties of the molecules contained in the *PA* extracts mentioned above, which counteract oxidative stress.

### 4.7. Correlation Analysis of Biochemical Parameters in HMM-Exposed Male Albino Rat Testis

This study investigates the interactive effects between antioxidant markers and oxidant, pro-inflammatory, transcriptional, and apoptotic biomarkers in the testes of rats exposed to the HMM and *PA*. Statistical analyses reveal a positive relationship between MDA and NF-kappa B, TNFα, and IL-6, suggesting an interaction associated with a protective effect on fecundity, consistent with the findings of Ozoani et al. [11]. Additionally, a positive correlation with GPx, CAT, and SOD, but a negative correlation with MDA, indicates evidence that the antioxidant system neutralizes ROS generated by the HMM in the testes and hinders inflammation and apoptosis. Taken together, these data unveil a nuanced response pattern induced by the administration of the HMM and *PA* [11].

### 4.8. Effect of Prosopis africana on Semen Analysis of Male Albino Rat Exposed to HMM

Metal treatments have been proven to affect the sperm quality in rats. In accordance with Ezejiofor and Orisakwe [102] and Adelakun and colleagues [103], we found a significantly lower sperm viability, a decrease in normal sperm count, and a significantly reduced overall sperm count in the HMM-treated group when compared to the control group. According to Barros and colleagues [104], oxidants appear to disrupt regular sperm activity by causing unsaturated fatty acid peroxidation in the sperm plasma membrane. Polyunsaturated fatty acids (PUFA), which are very vulnerable to oxidative damage from free radicals (ROS), cover mammalian spermatozoa. It is believed that the primary cause of ROS-induced sperm damage, which results in the loss of motility, aberrant morphology, decreased ability for sperm oocyte penetration, and infertility, is the lipid peroxidation (LPO) pathway [102]. In the treatment where a combination of the heavy metal mixture and *PA* aqueous extracts were administered, these observations were entirely lacking. This could be attributed to one of its main organic compounds, flavonoids, which are known to protect against oxidative injury by neutralizing oxygen radicals, preventing lipid peroxidation, and sequestering metal ions. Ultimately, this protects the sperm membrane, ensuring good quality sperm [56]. Recently, our histopathological studies on the architecture of the testis using a standard staining procedure (hematoxylin and eosin) [105] defined the grade of severity of oxidative damage of the HMM on spermatogenesis, confirming the gonado-protective role of *Prosopis africana* (see morphological evidence in Supplementary Materials Figure S1).

## 5. Conclusions

Taken together, this study highlighted the phytoconstituents detected in the Nigerian medicinal plant *Anonychium africanum* (*Prosopis africana*, *PA*) and their relevant activities. Experimental evidence indicates for the first time that the co-administration of the HMM with *PA* decreases oxido-inflammatory marker expression via the Nrf2 pathway, mitigating the deleterious gonadal effects of a heavy metal mixture and promoting male albino rat reproductive health. Therefore, our data on testicular oxidative stress, the expression of pro-inflammatory factors, and apoptotic and transcriptional factors demonstrate how the protective properties of *PA* are effective in alleviating testicular injuries induced by heavy metal mixture exposure, as evidenced by plasma reproductive hormone patterns and semen analysis. Studies incorporating a broader range of animal models (fish, amphibians, and reptiles) of both sexes to strengthen biodiversity sustainability are in progress; these include a large range of doses to provide a more comprehensive understanding of the treatment’s efficacy and safety over time. Further, mechanistic studies are currently underway to provide deeper insights into the molecular mechanisms of *PA* action.

## Figures and Tables

**Table 1:** The effect of the heavy metal mixture and *Prosopis africana* on feed intake, fluid intake, absolute testicular weight, relative testicular weight, and body weight (shown as initial weight, final weight, and percentage body weight difference).

Treatment	Feed Intake (g)	Fluid Intake (mL)	Absolute Testicular Weight (g)	Relative Testicular Weight (%)	Body Weight (g)		
					Initial Weight	Final Weight	% Body wt Difference
Control	164.75 ± 18.80 ^d^	225.08 ± 58.95 ^d^	3.18 ± 0.02 ^c^	1.1 ± 0.06 ^b^	175.0 ± 4.35	270.0 ± 13.73	54.29 ^a^
HMM	78.78 ± 27.54 ^a^	102.30 ± 20.03 ^a^	3.06 ± 0.32 ^a^	1.28 ± 0.39 ^a^	158.0 ± 2.00	240.0 ± 16.63	51.90 ^a^
HMM + *PA* (500 mg/kg)	88.53 ± 20.90 ^b^	148.10 ± 27.13 ^b^	2.84 ± 0.38 ^bc^	1.23 ± 0.08 ^b^	151.0 ± 1.00	230.3 ± 14.57	52.54 ^a^
HMM + *PA* (1000 mg/kg)	130.03 ± 18.48 ^c^	190.20 ± 56.50 ^c^	2.26 ± 0.11 ^bc^	1.06 ± 0.18 ^b^	146.0 ± 1.00	213.0 ± 27.15	45.89 ^a^
HMM + *PA* (1500 mg/kg)	158.55 ± 18.40 ^d^	218.31 ± 58.98 ^d^	2.01 ± 0.44 ^b^	0.96 ± 0.32 ^b^	141.0 ± 1.73	208.3 ± 20.81	47.75 ^a^

Values = Mean ± SD, N = 7. Values sharing the same letter notations (a, b, c, d) are not significantly different from each other (*p* ≥ 0.05); HMM = heavy metal mixture; *PA* = *Prosopis africana*.

**Table 2:** Effect of *Prosopis africana* on semen analysis of male albino rats exposed to heavy metal mixture.

Treatment	pH	Viable Cell Count (×10^6^ cells/mL)	Viscosity	Sperm Morphology (%)		Sperm Motility (%)			Sperm Count (×10^6^ cells/mL)
				Normal	Abnormal	Active	Sluggish	Immotile	
Control	8.5 ± 0.06 ^a^	0.85 ± 0.05 ^a^	Slightly viscous	85 ± 5 ^a^	15 ± 5 ^b^	82 ± 4 ^a^	8 ± 2 ^b^	10 ± 1 ^d^	716.7 ± 104.1 ^a^
HMM	8.1 ± 0.03 ^a^	0.61 ± 0.07 ^c^	Non viscous	68 ± 7 ^c^	31 ± 7 ^a^	58 ± 2 ^c^	11 ± 2 ^a^	30 ± 0 ^a^	266.7 ± 115.5 ^d^
HMM + *PA* (500 mg/kg)	8.1 ± 0.09 ^a^	0.70 ± 0.08 ^a^	Non viscous	73 ± 5 ^b^	26 ± 5 ^a^	70 ± 8 ^b^	11 ± 2 ^a^	18 ± 7 ^a^	383.3 ± 76.4 ^c^
HMM + *PA* (1000 mg/kg)	8.3 ± 0.02 ^a^	0.78 ± 0.02 ^b^	Non viscous	75 ± 10 ^b^	25 ± 10 ^a^	63 ± 5 ^c^	10 ± 1 ^a^	26 ± 5 ^a^	600.0 ± 173.2 ^b^
HMM + *PA* (1500 mg/kg)	8.5 ± 0.04 ^a^	0.85 ± 0.05 ^a^	Non viscous	83 ± 5 ^a^	15 ± 5 ^b^	85 ± 5 ^a^	8 ± 5 ^b^	8 ± 2 ^c^	733.3 ± 57.8 ^a^

Values = Mean ± SD, N = 7 Values sharing the same letter notations (a, b, c, d) are not significantly different from each other (*p* ≥ 0.05); HMM = heavy metal mixture; *PA* = *Prosopis africana.*

## Data Availability

Data is contained within the article or Supplementary Material.

## Supplementary Materials

The following supporting information can be downloaded at: https://www.mdpi.com/article/10.3390/antiox13091028/s1, Figure S1: Effect of heavy metal mixture (HMM) and co-administration of aqueous extract of the Nigerian medicinal plant *Anonychium africanum* (*Prosopis africana*, PA—1500 mg/kg) on albino rat testicular histoarchitecture.
