# Supplementary material for: Role of Anonychium africanum (Plantae, Fabaceae) in Metal Oxido-Inflammatory Response: Protection Evidence in Gonad of Male Albino Rat
Source: Antioxidants (Basel). 2024 Aug 24;13(9):1028. doi: 10.3390/antiox13091028 (PMC11429019; doi:10.3390/antiox13091028)
Supplement: Supplementary file 1 [file antioxidants-13-01028-s001.zip › antioxidants-3144599-supplementary.pdf]

antioxidants

MDPI

Article

Role of *Anonychium africanum* (Plantae, Fabaceae) by metal oxido-inflammatory response: protection evidence in gonad of male albino rat

Harrison A. Ozoani [1,†], Orish Ebere Orisakwe [1,2,†], Costantino Parisi [3], Loredana Assisi [3], Anthonet N. Ezejiofor [4], Kenneth O. Okolo [4], Chinna N. Orish [5], Rubina Vangone [3], Emidio M. Sivieri [3], and Giulia Guerriero [3,6,*]

Effect of heavy metal mixture (HMM) and co-administration of aqueous extract of the Nigerian medicinal plant, Anonychium africanum (Prosopis africana, PA - 1500 mg/kg) on albino rat testis histoarchitecture

MATERIALS and METHODS

The testis from all groups was excised, fixed in 10% formalin, dehydrated in a graded series of ethanol, and embedded in paraffin. The samples then underwent standard processing procedures. Briefly, paraffin-embedded testicular tissue samples were sectioned to a thickness of 4-5 μm, mounted on slides, and stained with hematoxylin and eosin (H&E) for histopathological examination. Signs of inflammation, fibrosis, necrosis, degeneration, and hypospermatogenesis were assessed.

RESULTS & DISCUSSION

The testis sections from the control condition (deionized H_2O only, Figure S1A) and PA only (data not shown) depict well-defined structures: seminiferous tubule (ST), interstitium (IS), and tunica albuginea (TA). It is appreciable that the testis exhibits a fully developed and normal germinal epithelium, featuring Leydig cells (LC), spermatogonia (SG), Sertoli cells (SC), primary spermatocytes (PS), secondary spermatocytes (SS), and spermatids (SM) and sperms (S) (Figure S1B).

Testis with HMM exposure are characterized by degeneration, as evidenced by the reduction of spermatids in multiple seminiferous tubules (Figure S1C). Specifically, inflammation, fibrosis, necrosis, and degeneration are observed in the organization of the germinal epithelium, with the exfoliation of spermatocytes (Figure S1D, arrow).

Testis exposed to simultaneous exposure to HMM and PA aqueous extracts at the highest concentration of PA (1500 mg/kg) are characterized by partial organized arrangements of all germ cells from spermatogonia to sperms in the seminiferous tubules (Figure S1E). Details of this section reveal regeneration, characterized by organized tubular arrangements and mature germ cells (sperms) in the seminiferous tubule, as depicted in Figure S1F.

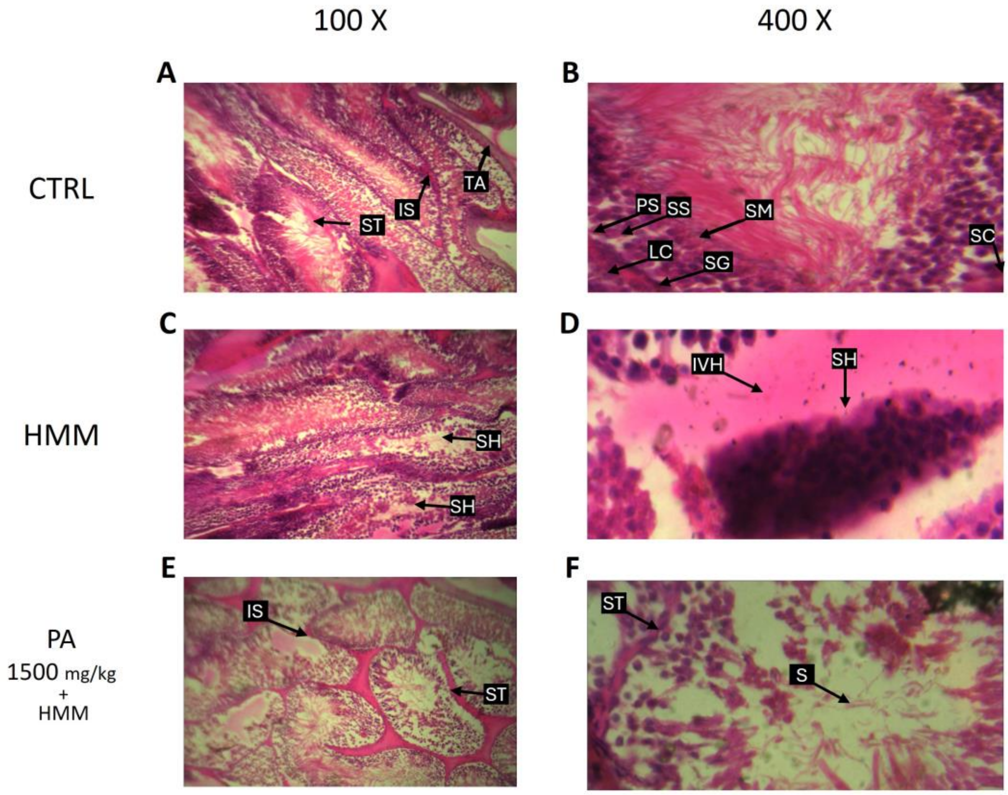

Figure S1. Histopathological photomicrographs for rat testis spermatogenesis on *Prosopis africana* (PA) treatment following exposure to heavy metal mixture (HMM) and 1500 mg/kg of PA at 100X and 400X. The legend: seminiferous tubule (ST), interstitium (IS), tunica albuginea (TA), Leydig cells (LC), spermatogonia (SG), Sertoli cell (SC), primary spermatocyte (PS), secondary spermatocyte (SS), spermatid (SM), sperm (S) interstitial vascular hemorrhage (IVH), intra-seminiferous hemorrhage (SH).
